# Transtibial Pullout Repair of Lateral Meniscus Posterior Root Tear with Tissue Loss: A Case with Anterior Cruciate Ligament Injury and Medial Meniscus Tear

**DOI:** 10.1155/2022/9776388

**Published:** 2022-08-31

**Authors:** Masanori Tamura, Takayuki Furumatsu, Takaaki Hiranaka, Keisuke Kintaka, Naohiro Higashihara, Yusuke Kamatsuki, Eiji Nakata, Toshifumi Ozaki

**Affiliations:** Department of Orthopaedic Surgery, Okayama University Graduate School of Medicine, Dentistry and Pharmaceutical Sciences, 2-5-1 Shikatacho, Kitaku, Okayama 700-8558, Japan

## Abstract

Lateral meniscus (LM) posterior root tear (LMPRT) is mainly caused by trauma, especially trauma associated with anterior cruciate ligament (ACL) injuries. Although a transtibial pullout repair or a side-to-side repair is commonly performed for LMPRT, to the best of our knowledge, there is no clinical report of LMPRT with tissue loss using the pullout technique. Thus, the purpose of this report was to describe a clinical, radiographic, and arthroscopic outcome after pullout repair for a case of LMPRT with a large defect with a chronic ACL tear and complex medial meniscus (MM) tears. A 31-year-old man complained of knee pain and restricted range of motion after twisting his knee when he stepped on an iron pipe. The patient had a football-related injury to his right knee 14 years before presentation, and since then, the patient's knee has given out more than 10 times but was left unassessed. Magnetic resonance imaging showed LMPRT with tissue loss, ACL tears, and complex MM tears. Transtibial pullout repair of the LMPRT with ACL reconstruction and MM repairs were performed. Following the pullout repair of the LMPRT, an approximately 6 mm gap remained between the LM posterior root and root insertion. However, magnetic resonance imaging and second-look arthroscopy at 1 year postoperatively revealed meniscal healing, gap filling with some regeneration tissue, of the LM posterior root. Furthermore, the lateral meniscus extrusion in the coronal plane improved from 3.1 mm (preoperative) to 1.6 mm (1 year postoperatively). Transtibial pullout repair with the remaining gap could be a viable treatment option for LMPRT with tissue loss, combined with ACL reconstruction.

## 1. Introduction

Meniscal root tears are defined as radial tears located within 1 cm of the meniscal root insertion or an avulsion of the insertion of the meniscus. The incidence rate of lateral meniscus (LM) posterior root tear (LMPRT) combined with anterior cruciate ligament (ACL) tear is reported to be around 6.6–14% [1]. Repair of LMPRT is recommended as possible and supported by biomechanical studies for mainly two reasons: first, a meniscal root tear injury typically results in the loss of meniscal circumferential hoop stress and can lead to meniscal extrusion [2], decreased tibiofemoral contact surface [3], increased cartilage stress, and ultimately to articular degeneration; second, LMPRT contributes to rotational instability in patients who are ACL-deficient, as confirmed by an increase in anterior tibial translation of the ACL-deficient knee during a pivot shift maneuver [4, 5].

Although extensive clinical studies are lacking, a recent systematic review reported favorable functional scores following ACL reconstruction and LMPRT repair [6]. The pullout repair is one of the most commonly performed techniques in root avulsion and radial tears. However, to the best of our knowledge, no clinical report of LMPRT with tissue loss using the pullout technique and the gap healing of the defect has been previously reported.

Here, we describe the surgical treatment, healing status, and clinical results of a case of LMPRT with a tissue loss concomitant with a chronic ACL tear and complex medial meniscus (MM) tears.

## 2. Case Presentation

A 31-year-old male, currently working as a long-distance driver, injured his right knee by stepping on an iron pipe, resulting in his knee twisting. The patient had also injured his right knee while playing football 14 years before presentation, for which he received conservative management. Since then, the patient's knee gave out more than 10 times but was left unassessed.

The patient's chief complaint was right-knee pain, limited range of motion in the knee, and painful movement. The Lachman test and pivot shift test yielded positive results. Preoperative radiographs were normal, and the posterior tibial slope was 7°. Magnetic resonance imaging (MRI) revealed an ACL rupture, an LMPRT concomitant with LM extrusion, and MM tears.

**Figure 1:**
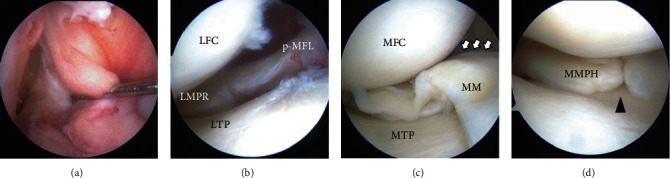
(a–d) Arthroscopic findings. (a) Chronic ACL tear. (b) The torn edge of the LMPR was defected. (c) Reduced MM bucket handle tear (white arrows) extending from the body to the posterior horn. (d) Complete radial tear of the posterior horn of the MM (black arrowhead). Abbreviations: LTP: lateral tibial plateau; LFC: lateral femoral condyle; p-MFL: posterior meniscofemoral ligament (Wrisberg ligament); MTP: medial tibial plateau; MFC: medial femoral condyle; MMPH: medial meniscus posterior horn.

An arthroscopic evaluation revealed that the ACL was torn, with only the scar tissue remaining (Figure 1(a)). The LM posterior root (LMPR) was detached and defected about more than 12 mm (Figure 1(b)). The posterior meniscofemoral ligament was intact. After reduction of the displaced MM, the bucket handle tear of the MM extended from the body to the posterior horn (Figure 1(c)). The complete radial tear at the posterior horn of the MM extended from the central to the peripheral lesion near the bucket handle tear (Figure 1(d), supple Fig. 1).

### 2.1. MM Repair

**Figure 2:**
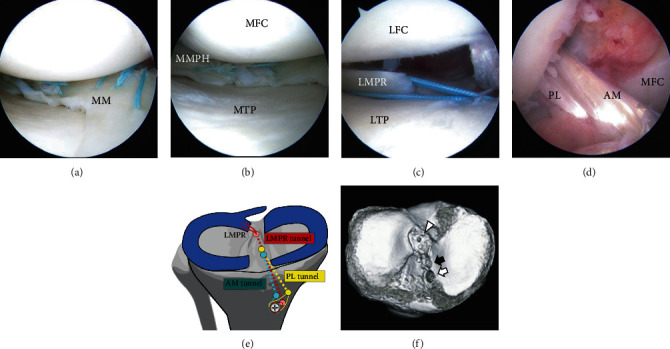
(a–d) Arthroscopic findings and illustrations of the tunnel positions and postoperative three-dimensional computed tomography. (a) Repaired bucket handle tear of the MM. (b) Repaired radial tear of the posterior horn of the MM. (c) A 6 mm gap between the edge of the repaired LMPR and the bone aperture site remained. (d) Reconstructed ACL. (e) Illustration of the tunnel locations for the pullout repair of the LMPRT and the reconstruction of the AM bundle and the PL bundle. (f) The tibial tunnel for the LMPRT (arrowhead) was placed separately with a tunnel for the PL bundle (black arrow) and the AM bundle (white arrow).

We first implemented the outside-in pie-crusting technique using a standard 18-gauge (1.2 × 40 mm) hypodermic needle (TERUMO, Tokyo, Japan) to increase the medial joint space. After reduction of the displaced MM into its appropriate anatomic position, 6 sutures were performed using 2-0 Wayolax (Matsuda Ika Kogyo, Tokyo, Japan) using the inside-out technique in the vertically stacked way for the bucket handle tear (Figure 2(a)). Following the repair of the bucket handle tear, a cross-stitch with two perpendicular sutures in an “X” configuration was performed using 2-0 Wayolax sutures using inside-out technique for the radial tear (Figure 2(b)) [7].

### 2.2. LMPR Repair

We performed a transtibial pullout repair for LMPRT using two simple stitches. A Knee Scorpion suture passer (Arthrex, Naples, Florida, USA) was used to pass two No. 2 Ultrabraid™ sutures (Smith & Nephew, Andover, Massachusetts, USA) vertically through the meniscal tissue. We made an additional bone tunnel in the center of the root attachment, which was about 4 mm medial and 1.5 mm posterior from the lateral tibial eminence, as previously reported by Johannsen et al. in a cadaveric anatomical study [8]. A 2.4 mm guide pin was inserted using an Acufex Director Elite™ ACL drill guide system (Smith & Nephew), aiming at the center of the root attachment. The sutures were retrieved through the additional tunnel and tied under the 5.0 mm cancellous screw inserted at the tibia with the knee flexed at 20° with an initial tension of 20 N after ACL reconstruction. Following the procedure, an approximately 6 mm gap between the edge of the LMPR and the root insertion site remained (Figure 2(c)).

### 2.3. ACL Reconstruction

We performed double-bundle reconstructions with semitendinosus autografts using the outside-in technique. The femoral tunnel was created using an anterolateral portal entry femoral aimer and an Acufex Director Elite™ ACL drill guide system. Femoral tunnels were created with a 6 mm diameter reamer for the anteromedial (AM) bundle and a 5.5 mm diameter reamer for the posterolateral (PL) bundle. The tibial aiming guide was set at 45° for the AM bundle and 55° for the PL. Tibial tunnels were created with a 6.0 mm diameter reamer for the AM and PL bundles. The two bundles were passed from the tibial tunnel to the femoral tunnel through two leading sutures using the EndoButton CL device (Smith & Nephew) (Figure 2(d)). The tibial fixation was performed with the knee flexed at 20°, with an initial tension of 30 N for the AM bundle and in full extension with 20 N for the PL bundle. These bundles were fixed using the tenodesis screw (Arthrex), and the passing sutures were tied under the 5.0 mm cancellous screw, similar to the repair suture for LMPRT (Figure 2(e)). The aperture location of the tibia was confirmed by computed tomography examination after the surgery (Figure 2(f)).

### 2.4. Postoperative Protocol

The patient was initially nonweight-bearing with a limited-motion knee brace. At 3 weeks postoperatively, 1/3 partial weight-bearing was permitted, with progression to full weight at 5 weeks postoperatively. The patient was allowed 90° knee flexion at 2 weeks postoperatively, 120° at 3 weeks postoperatively, and deep flexion at 5 weeks postoperatively. Jogging was allowed following a muscle strength check at 4 months postoperatively. Sports activities were allowed at 9 months postoperatively.

### 2.5. Clinical Assessment and Second-Look Arthroscopy

**Figure 3:**
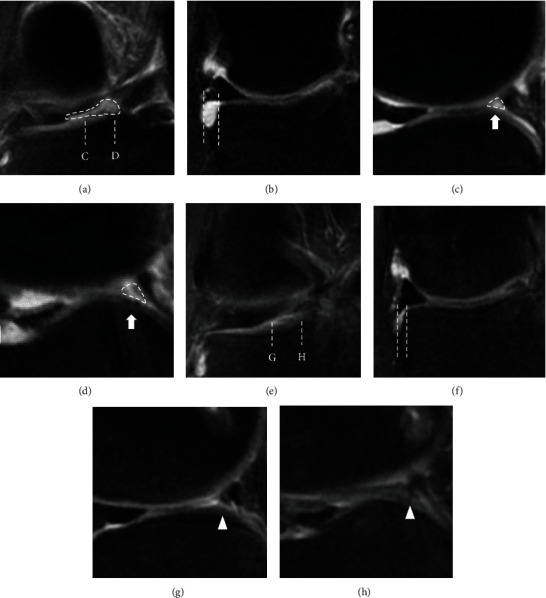
Comparison of the (a–d) preoperative and (e–h) postoperative MRI findings. (a) Preoperative MRI showed a linear defect in the coronal plane (dotted area). (b) The length of the lateral meniscus extrusion (LME) was 3.1 mm (length between dashed lines). (c, d) The reference lines of the sagittal plane are shown as dotted lines in (a) (C and D, respectively). Ghost sign in two consecutive sagittal planes with a 10 mm interval (dashed area). (e) One year after the operation, the preoperative gap was filled with tissue continuous to the tibial attachment of the LMPR in the coronal plane. (f) The length of LME was 1.6 mm (1 year postoperatively). (g, h) The reference lines of the sagittal plane are shown as dotted lines in (e) (G and H, respectively). The disappearing ghost sign in two consecutive sagittal planes with a 10 mm interval (arrowhead).

**Figure 4:**
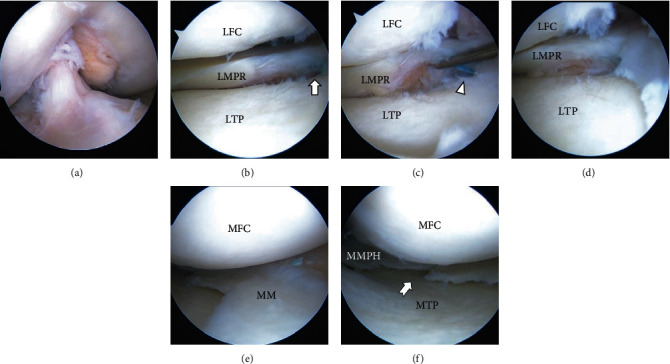
Arthroscopic findings during the second-look arthroscopy. (a) Reconstructed ACL. (b) The repaired LMPR healed with continuity to its insertion site (arrow). (c) The repair sutures were cut out (arrowhead). (d) The synovial coverage around the root attachment. (e) The repaired MM bucket handle tear completely healed. (f) An approximately 1 cm defect remained at the central margin of the repaired radial tear (swallow-tail arrow).

Preoperative MRI of the coronal plane revealed the tissue loss of the LMPRT (Figure 3(a)), and the length of the LM extrusion (LME) was 3.1 mm (Figure 3(b)). The tissue loss of LMPRT was also found in sagittal planes with a 10 mm interval as a ghost sign (Figures 3(c) and 3(d)). MRI and the second-look arthroscopy were performed 1 year postoperatively. The one-year postoperative MRI of the coronal plane revealed continuous tissue regeneration, and the LME improved to 1.6 mm (Figures 3(e) and 3(f)). The regeneration tissue was also found in postoperative MRI of sagittal planes with a 10 mm interval as a disappearing ghost sign. The regeneration tissue might be small; the signal intensity was slightly high in T2 fat saturation serials (not similar in signal to the normal meniscus) but had a triangular shape in the sagittal plane (Figures 3(g) and 3(h)). Second-look arthroscopy at 12 months postoperatively revealed that the maturation of the reconstructed ACL graft was good, with good graft integrity, tension, and synovial coverage (Figure 4(a)). The repaired LMPR healed with continuity to the root attachment (Figures 4(b)–4(d)), although the repair sutures were cutout (Figure 4(c)), and synovial coverage around the root attachment was found (Figure 4(d)). The MM bucket handle tear was completely healed (Figure 4(e)); however, an approximately 1 cm defect remained at the central margin of the radial tear, needing marginal debridement (Figure 4(f)). The side-to-side difference of the anterior tibial translation measured by a KT-2000 arthrometer was not found in both knees. At the final follow-up performed 2 years after the first operation, the clinical outcome had improved compared to the preoperative status (Table 1).

## 3. Discussion

An important finding in this case report was that the LMPRT with a tissue loss was successfully repaired through transtibial pullout repair of LMPRT concomitant with the ACL reconstruction. Second-look arthroscopy revealed the healing of the LMPRT, and MRI showed LME reduction 1 year following the procedure.

In this case, the devastating LM and MM injury could be related to ACL deficiency. LMPRTs most frequently occur in an acute ACL-ruptured knee [1, 9, 10], and an LMPRT tissue loss could result from chronicity [11]. An anterior tibial translation and a squeeze of the LM between the femur and the tibia have been reported as possible pathology for LMPRT [9]. An MM bucket handle tear is often accompanied by various ACL injuries [12]. We planned to perform suture repairs of all the tears because all tears influence knee kinematics and meniscal function, including shock absorption and load distribution within the knee. An ACL is the most powerful static stabilizer and is crucial for anteroposterior and tibial internal rotatory stability at a lower flexion angle [13]. An LMPR contributes to rotational stability in patients who are ACL deficient [4, 5]. Moreover, a full-thickness MM radial tear renders the meniscus dysfunction from loss of hoop stress resistance, which is biomechanically comparable to a complete meniscectomy [14].

Following LMPRT repair, an approximately 6 mm gap remained between the retracted LMPR and the tibial tunnel aperture; nevertheless, second-look arthroscopy 1 year after the operation showed the healing of the LMPR lesion. Some possible positive factors related to this gap healing were knee stability obtained through ACL reconstruction, vascularity, and marrow content. Generally, meniscal tissue is neutralized via diffusion from blood vessels and convection from the synovial fluid of the joint cavity. The tendon-like collagenous connective tissue of the posterior horn attachment sites is vascularized regardless of age [15]. Moreover, the marrow content, including mesenchymal stem cells from the opened bone tunnel, may enhance the healing process [16]. Other possible factors include structural stability related to the integrity of the meniscofemoral ligament, the graft or the repaired meniscus tension [17], and the meniscal position. The repaired connective tissue from the retracted LMPR and the root attachment appeared thinner than the intact meniscus during the second-look arthroscopy. However, histological analysis of the repaired tissue was not performed. Furthermore, we could not compare our findings with those of other studies because of the lack of reports about gap healing.

We created an additional tibial tunnel for the LMPRT repair instead of using the tibial tunnel for the PL bundle (Figure 2(e)). Some reports recommend the transtibial pullout repair of the LMPRT using the tibial PL tunnel [10, 18] because it is an anatomical repair and is minimally invasive and eliminates the necessity for additional bone tunnel creation [3, 10]. However, in our case, the LMPR was positioned too far in the anterior direction when we first pulled out the suture from the tibial PL tunnel. The anatomical repair of the meniscal root is important for its function in converting femorotibial loads into circumferential tension [19]. We, therefore, drilled the additional tunnel aiming at the posteromedial direction from the lateral tibial eminence and anterior from the PCL, as previously described [8, 20]. One concern regarding the additional tunnel was tunnel convergence with ACL tunnels [21]; however, in our case, only a 2.4 mm tunnel was needed to pass the suture, and enough margin had been confirmed (Figure 2(f)). Our technique was simple, easy to perform, and not time-consuming.

Previous retrospective studies have revealed that LME is related to LMPRT in patients with ACL deficiency [2]. Some recent reports have used MRI findings of LME as an objective indicator for measuring postsurgical meniscal function after LMPR repair concomitant with ACL reconstruction [11, 22]. Ahn et al. [11] and Tsujii et al. [22] reported satisfactory clinical results after side-to-side repair for LMPRT concomitant with ACL reconstruction without LME reduction confirmed using coronal plane MRI images taken after the operation. On the other hand, Okazaki et al. reported that the transtibial pullout repair successfully decreased LME when compared to other techniques, such as all-inside repair and inside-out repair [23]. Furthermore, Zhuo et al. reported a linear relationship between postoperative LME and increased progression of cartilage degeneration at the second-look arthroscopy after the pullout repair of LMPRT [24]. In our case, the LME in the coronal plane improved from 3.1 mm (preoperative) to 1.6 mm at the final MRI follow-up (1 year postoperatively). Since extensive clinical studies are lacking, it is not clear in our case whether the residual LME of 1.6 mm is enough to slow down the progression of cartilage degeneration. However, if the LMPRT is left untreated during ACL reconstruction, the lateral joint-space narrowing of 1.0 mm is reported at a mean follow-up of 10 years [25]. We believe that the pullout repair of the LMPRT is beneficial for restoring meniscus function and could be available to treat even tissue loss of the LMPRT at the same time as ACL reconstruction in younger patients.

Here, we report a case of LMPRT with a large tissue loss with a chronic ACL tear and complex MM tears. Despite our patient having devastating LM and MM tears with ACL deficiency, favorable meniscal healing and clinical outcomes were achieved. In conclusion, transtibial pullout repair for LMPRT could effectively treat LMPRT with a tissue loss concomitant with ACL reconstruction.

## Figures and Tables

**Table 1:** Clinical and radiographic findings recorded preoperatively and at final follow-up (2 years after the first operation).

	Preoperative	Final follow-up
Lysholm score	65	90
Tegner activity scale	2	4
IKDC score	39	92
KOOS		
Pain	44	86
Symptoms	50	91
Activities of daily living	60	100
Sport and recreation	10	85
Knee-related QOL	44	81
Total	48	91
VAS	13	2
Kellgren and Lawrence classification (right/left)	1/1	1/1

IKDC: International Knee Documentation Committee; KOOS: Knee Injury and Osteoarthritis Outcome Score; QOL: quality of life; VAS: visual analog scale.

## Data Availability

Data is available on request.

## Supplementary Materials

Supplementary Materials Supplemental Figure 1: the schematic description of the right tibial plateau from above, showing the LMPRT and complex MM tears.
