# Supplementary material for: Transtibial Pullout Repair of Lateral Meniscus Posterior Root Tear with Tissue Loss: A Case with Anterior Cruciate Ligament Injury and Medial Meniscus Tear
Source: Case Rep Orthop. 2022 Aug 31;2022:9776388. doi: 10.1155/2022/9776388 (PMC9453023; doi:10.1155/2022/9776388)
Supplement: Supplementary Materials — Supplemental Figure 1: the schematic description of the right tibial plateau from above, showing the LMPRT and complex MM tears. [file 9776388.f1.pdf]

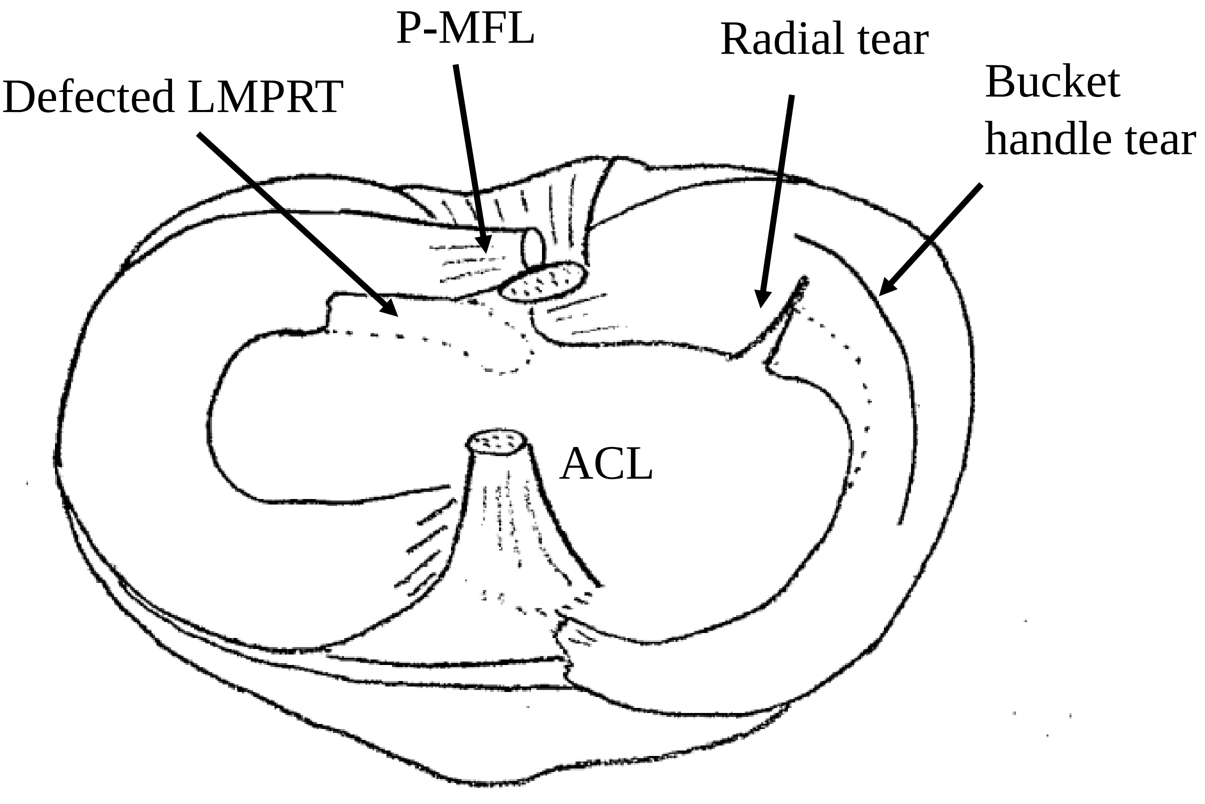

Supplemental Fig. 1. The schematic description of the right tibial plateau from above, showing the LMPRT and complex MM tears.

Supplemental Fig.1
